# Functional Variants in *Linc-ROR* are Associated with mRNA Expression of *Linc-ROR* and Breast Cancer Susceptibility

**DOI:** 10.1038/s41598-018-22881-x

**Published:** 2018-03-16

**Authors:** Chenglin Luo, Jingjing Cao, Rui Peng, Qiaoyun Guo, Hua Ye, Peng Wang, Kaijuan Wang, Chunhua Song

**Affiliations:** 1 Department of Epidemiology and Statistics, College of Public Health, Zhengzhou University, Zhengzhou, 450001 Henan PR China; 2 Department of preventive medicine, Heze Medical College, Heze, 274000 Shandong China; 3 Department of Biological Sciences, The University of Texas at El Paso, TX, 79968 USA

## Abstract

Functional polymorphisms in *Linc-ROR* may change its ability of regulation by regulating *Linc-ROR* expression. However, these functional polymorphisms in *Linc-ROR* and their associations with breast cancer (BC) susceptibility were scarcely reported. In this molecular epidemiological study, four SNPs (rs6420545, rs4801078, rs1942348 and rs9636089) were selected in *Linc-ROR* by bioinformatics method. Unconditional logistic regression model was performed to analyze the associations between four SNPs and BC susceptibility adjusted for reproductive factors. Quantitative real-time (qRT) PCR was used to evaluate relative expression of *Linc-ROR* in plasma. The interactions of gene reproductive factors were assessed by Multifactor Dimensionality Reduction (MDR) method. A novel finding showed TT (*OR*: 1.79; *95%CI*: 1.20–2.68) genotype of rs4801078 in *Linc-ROR* had a significant association with the higher risk of BC and the expression of *Linc-ROR* mRNA was closely related with the alleles of rs4801078. In addition, we found the interaction of rs4801078, number of pregnancy and menopausal status might increase BC risk (*OR*: 2.78; *95%CI*: 2.74–3.61). Our results suggest that interactions of SNPs in *Linc-ROR* and reproductive factors might contribute to BC risk, and alleles of rs4801078 might affect *Linc-ROR* expression level.

## Introduction

Breast cancer is the most common diagnosed cancer and the leading cause of cancer death in Chinese women^1^. It alone is expected to account for fifteen percent of all new cancers in women with about 4292,000 newly diagnosed invasive cancer cases in 2015 in China^2^.

Noncoding RNAs (ncRNAs) which function by means other than directing the production of proteins were a distinguishing feature of metazoan genomes^3^. Numerous studies have underlined the regulatory role of microRNAs (miRNAs) in the development of cancers, and their variants are reported to be related to various cancer^4–6^. LncRNAs ranging from 200 nucleotides (nt) to over 10 kb are spliced, polyadenylated, and are roughly as diverse in a given cell type as protein-coding mRNAs^7–10^. In addition to the small regulatory RNAs, emerging studies indicate that lncRNAs play critical roles in various biological processes ranging from embryonic development to human diseases, including controlling cell cycle progression, apoptosis, invasion, and migration^11^. The aberrant expressions of several lncRNAs in various cancers indicate that lncRNAs may be play roles in tumor carcinogenesis^12–14^. Moreover, recent studies have shownthe important roles of lncRNAs and their genetic variants played in tumor carcinogenesis. Yan *et al*. found that TC genotype of rs10463297 in lncRNA SRA could increase BC risk compared with CC genotype^15^. Reactivation of the H19 gene has been observed in bladder tumors, and TC genotype of rs2839698 in H19 was found to decrease bladder cancer risk^16^. Yao *et al*. suggested that individuals with rs7958904 CC genotype in *HOTAIR* had asignificantlydecreasedriskofcolorectalcancer^17^.

Large intergenic noncoding RNA regulator of reprogramming (*lincRNA-ROR*, *Linc-ROR*), at 2.6 kb in length, was first identified as a regulator for reprogramming of differentiated cells to induced pluripotent stem cells (iPSCs)^18^. *Linc-ROR* also functions as a microRNA sponge to prevent the core transcription factors (TFs) Oct4, Nanog, and Sox2 from miRNA-mediated suppression in self-renewing human embryonic stem cells (ESCs)^19^ and studies indicated that *Linc-ROR* could act as a p53 repressor in response to DNAdamage^20^. All the evidence intrigued us to speculate that *Linc-ROR* might also have a role in cancer progression and we found several studies have focused the role of *Linc-ROR* in the development of cancers, including breast cancer^21–24^.

Reproductive factors are closely related to the development of breast cancer and the research on interaction between reproductive factors and susceptibility gene has been done^25^. However, up to now, research combining the SNPs in *Linc-ROR* and reproductive factors on BC risk has not been reported. In our current case-control study, we selected four tagger SNPs (rs6420545, rs4801078, rs1942348 and rs9636089) in *Linc-ROR* by bioinformatics method and explored a potential correlation between those potentially functional polymorphisms and risk of BC in Chinese women. Furthermore, analysis of qRT - PCR was applied to detect the relative expression level of *Linc-ROR* in plasma and the interactions of gene reproductive factors were analyzed using MDR method.

## Results

### Characteristics of patients and controls

The demographic and clinical characteristics of 968 subjects were displayed in Table 1. The mean age of patients and matched controls were 48.41 ± 10.21 and 49.23 ± 10.15 respectively (*P* = 0.21). The mean age at menarche of BC patients (14.48 ± 1.82) was significantly higher than mean age at menarche of controls (14.08 ± 1.78). More than 4 pregnancies for women increased the risk of BC (*OR*: 2.01, *95%CI*: 1.32–3.06). However, there is no difference in the distributions of the other characteristics.

**Table 1:** Thebaseline characteristics of total 484 BC patients and 484 cancer-free controls.

Variables	Cases (%)	Controls (%)	*P* ^b^	*OR(95%CI)*
	n = 484	n = 484		
Age (Mean ± SD)	48.41 ± 10.21	49.23 ± 10.15	0.211^a^	
Age at menarche (Mean ± SD)	14.48 ± 1.82	14.08 ± 1.78	**0**.**001**^a^	
Age at menopause				
≤50	352 (0.73)	337 (0.70)		1
>50	132 (0.27)	147 (0.30)	0.287	0.86 (0.65–1.14)
Menopausal state				
Pre-menopausal	291 (0.60)	283 (0.59)		1
Post-menopausal	193 (0.40)	201 (0.41)	0.601	0.93 (0.72–1.21)
No. of abortions				
≤1	336 (0.69)	330 (0.68)		1
2~3	123 (0.25)	138 (0.29)	0.382	0.87 (0.65–1.18)
≥4	25 (0.06)	16 (0.03)	0.189	1.59 (0.80–3.16)
No. of pregnancy				
≤1	60 (0.12)	87 (0.18)		1
2	116 (0.24)	156 (0.33)	0.727	1.08 (0.70–1.66)
3	132 (0.27)	114 (0.24)	**0**.**019**	**1**.**68** (**1**.**09–2**.**60)**
≥4	176 (0.37)	127 (0.26)	**0**.**001**	**2**.**01** (**1**.**32–3**.**06)**
Breast-feeding				
No	27 (0.06)	42 (0.09)		1
Yes	457 (0.94)	442 (0.91)	0.063	1.61 (0.98–2.65)
Family history				
No	462 (0.95)	463 (0.96)		1
Yes	22 (0.05)	21 (0.04)	0.876	1.05 (0.57–1.94)
ER				
Negative	149 (0.31)			
Positive	293 (0.61)			
PR				
Negative	188 (0.39)			
Positive	253 (0.52)			
HER-2				
Negative	120 (0.25)			
Positive	308 (0.64)			

^a^Student’s t test.

^b^Two-sided *χ*^2^ test, *P* < 0.05 was considered to be statistically significant.

### The SNPs genotypes were associated with BC

Table 2 showed the distributions of genotypes in BC patients and control group for four selected SNPs. All of SNPs genotypes in control group meet Hardy-Weinberg equilibrium (*P* = 0.99 for rs1942348, 0.77 for rs4801078, 0.94 for rs6420545 and 0.99 for rs9636089). TT (*OR*: 1.79; *95%CI*: 1.20–2.68) genotype of rs4801078in *Linc-ROR* increased BC risk incodominant model. Also, TT (*OR*: 1.84, *95%CI*: 1.28–2.66) genotype of rs4801078 in *Linc-ROR* sho wed increased risk of BC in recessive model.

**Table 2:** The association between four SNPs genotypes and risk of breast cancer.

SNPs	Genetic model	Genotype	Case (%)	Control (%)	*P* ^a^	Adjusted *OR(95%CI)*^b^	*P* ^b^
			n = 484	n = 484			
rs1942348					0.99		
	Codominant	TT	184(0.38)	183(0.38)		1	
		CT	227(0.47)	227(0.47)		0.91 (0.67–1.23)	0.529
		CC	73(0.15)	74(0.15)		0.99 (0.65–1.51)	0.959
	Dominant	TT				1	
		CT + CC	300(0.62)	301(0.62)		0.93 (0.70–1.23)	0.602
	Recessive	TT + TC	411(0.85)	410(0.85)		1	
		CC				1.04 (0.71–1.54)	0.827
	Over-dominant	TT + CC	257(0.53)	257(0.53)		1	
		TC				0.91 (0.69–1.20)	0.507
	Allele	T	595(0.61)	593(0.61)		1	0.981
		C	373(0.39)	375(0.39)		1.01(0.83–1.20)	
rs4801078					0.77		
	Codominant	CC	162(0.33)	176(0.36)		1	
		CT	211(0.44)	238(0.49)		0.95 (0.70–1.30)	0.740
		TT	111(0.23)	70(0.15)		**1**.**79(1**.**20–2**.**68)**	**0**.**005**
	Dominant	CC				1	
		CT + TT	322(0.67)	308(0.64)		1.14 (0. 85–1.52)	0.386
	Recessive	CC + CT	373(0.77)	414(0.85)		1	
		TT				**1**.**85 (1**.**28–2**.**66)**	**0**.**001**
	Over-dominant	CC + TT	273(0.56)	246(0.51)		1	
		CT				0.78 (0.59–1.02)	0.078
	Allele	C	535(0.55)	590(0.61)		1	**0**.**022**
		T	433(0.45)	378(0.39)		**1**.**24(1**.**03–1**.**48)**	
rs6420545					0.94		
	Codominant	CC	126(0.26)	120(0.25)		1	
		CT	228(0.47)	236(0.49)		0.88(0.63–1.24)	0.462
		TT	130(0.27)	128(0.26)		1.05 (0.72–1.54)	0.794
	Dominant	CC				1	
		CT + TT	358(0.74)	364(0.75)		0.94(0.68–1.29)	0.695
	Recessive	CC + CT	354(0.73)	356 (0.74)		1	
		TT				1.14 (0.83–1.57)	0.408
	Over-dominant	CC + TT	256(0.53)	248 (0.51)		1	
		CT				0.86 (0.65–1.13)	0.282
	Allele	C	480(0.50)	476(0.49)		1	0.518
		T	488(0.50)	492(0.51)		0.94(0.79–1.23)	
rs9636089					0.99		
	Codominant	TT	187(0.39)	191(0.39)		1	
		CT	223(0.46)	226(0.47)		0.90(0.66–1.21)	0.475
		CC	74(0.15)	67(0.14)		1.03 (0.67–1.58)	0.896
	Dominant	TT				1	
		CT + CC	297(0.61)	293(0.61)		0.93 (0.70–1.23)	0.596
	Recessive	TT + TC	410(0.85)	417(0.86)		1	
		CC				1.09(0.74–1.62)	0.661
	Over-dominant	TT + CC	261(0.54)	258(0.53)		1	
		TC				0.89 (0.67–1.18)	0.408
	Allele	T	597(0.62)	608(0.63)		1	0.680
		C	371(0.38)	360(0.37)		1.04(0.86–1.25)	

^a^*P*value of Hardy-Weinberg equilibrium in controls;

^b^*P* value of logistic regression analysis with adjusted for age, age at menarche, menopausal status, number of pregnancy and abortion, breast-feeding status, and family history of BC in first-degree relatives.

### Stratified analysis

We evaluated the relationship of SNPs genotypes and BC risk stratified by the reproductive factors (Table 3). The positive effect of rs6420545 CT + TT genotype was more significant in the subjects (*P* = 0.005) with age at menarche ≤13 (*OR*: 0.95, *95%CI*: 0.92–0.99) and rs19 CT + TT genotype were more evident in the subjects (*P* = 0.045) with age >50 (*OR*: 0.60, *95%CI*: 0.37–0.99). The protective role of CT + CC genotypes for rs9636089 were more obvious in the women (*P* = 0.021) with age > 50 (*OR*: 0.57, *95%CI*: 0.35–0.92).In addition, the CT + TT genotypes of rs4801078revealed a significant higher risk of BC in the participant with age >50 (*OR*: 2.14, *95%CI*: 1.33–3.45) and age of menarche >13 (*OR*: 1.52, *95%CI*: 1.06–2.19).

**Table 3:** Stratification analysis of the fiveSNPs and BC susceptibility.

variables	case	control	rs6420545		rs4801078		rs1942348		rs9636089	
			*OR (95%CI)* ^a^	*P* ^a^	*OR (95%CI)* ^a^	*P* ^a^	*OR (95%CI)* ^a^	*P* ^*a*^	*OR (95%CI)* ^a^	*P* ^a^
			TT vs CT + CC		CC vs CT + TT		CC vs CT + TT		TT vs CT + CC	
age										
≤50	302	285	0.70 (0.46–1.05)	0.083	0.79(0.54–1.14)	0.206	*1*.*16(0*.*81–1*.*67)*	*0*.*41*	1.24 (0.87–1.78)	0.238
>50	182	199	1.50(0.90–2.52)	0.125	2.14 (1.33–3.45)	**0**.**002**	*0*.*60(0*.*37–0*.*99)*	***0***.***045***	0.57(0.35–0.92)	**0**.**021**
Age at menarche										
≤13	152	201	0.95 (0.92–0.99)	**0**.**005**	0.69(0.42–1.13)	0.14	*1*.*38(0*.*85–2*.*26)*	*0*.*196*	1.19(0.74–1.93)	0.476
>13	332	283	1.46 (0.98–2.16)	0.061	1.52(1.06–2.19)	**0**.**025**	*0*.*77(0*.*54–1*.*11)*	*0*.*162*	0.83(0.58–1.19)	0.31
Age at menopause										
≤50	352	337	0.91(0.64–1.32)	0.630	1.16 (0.83–1.62)	0.4	*1*.*01(0*.*72-0*.*1*.*42)*	*0*.*946*	1.03(0.74–1.44)	0.85
>50	132	147	1.13 (0.58–2.19)	0.726	1.13(0.62–2.06)	0.68	*0*.*67(0*.*38–1*.*20)*	*0*.*18*	0.66 (0.37–1.16)	0.147
No. of pregnancy										
≤2	176	243	0.98 (0.58–1.66)	0.95	1.32 (0.82–2.12)	0.155	*0*.*96(0*.*61–1*.*52)*	*0*.*866*	0.86 (0.55–1.35)	0.512
>2	308	241	0.95(0.63–1.43)	0.807	*1*.*08 (0*.*74–1*.*59)*	*0*.*679*	*0*.*89(0*.*61–1*.*31)*	*0*.*577*	0.96(0.66–1.41)	0.848
No. of abortion										
≤2	417	438	0.95(0.68–1.33)	0.776	1.16 (0.86–1.57)	0.339	*0*.*91(0*.*68–1*.*24)*	*0*.*559*	0.93(0.69–1.25)	0.622
>2	67	46	1.10(0.44–2.73)	0.843	1.13(0.46–2.77)	0.784	0.86(0.37–2.02)	0.729	0.84(0.36–1.98)	0.691
Breast-feeding										
no	27	42	46.67 (2.46–884.21)	**0**.**010**	18.72(1.17–300.7)	**0**.**039**	0.56(0.16–1.96)	0.366	0.60(0.17–2.11)	0.422
yes	457	442	0.89 (0.64–1.23)	0.473	1.11 (0.82–1.49)	0.513	*0*.*95(0*.*71–1*.*28)*	*0*.*735*	0.94(0.70–1.27)	0.696
Family history										
no	462	463	1.01(0.73–1.40)	0.947	1.21(0.90–1.64)	0.212	*0*.*92(0*.*69–1*.*24)*	*0*.*599*	0.90 (0.67–1.21)	0.473
yes	22	21	0.29(0.05–1.55)	0.147	0.52(0.10–2.80)	0.448	0.65(0.14–2.92)	0.573	1.33 (0.31–5.77)	0.701

^a^*P*value of logistic regression analysis with adjusted for age, age at menarche, menopausal status, number of pregnancy and abortion, breast-feeding status, family history of BC in first-degree relatives(the stratified factor in each stratum excluded).

### The association of receptor status and the four SNPs genotypes

The association between SNPs in *Linc-ROR* and the receptors status in cases were displayed in Table 4. After adjusted for reproductive factors, only a boundary significant association between rs6420545 CT (*OR*: 0.66; *95%CI*: 0.99–2.76) genotype with PR status of patients was detected.

**Table 4:** The Associations between four SNPs and ER, PR and HER-2 Status of Breast Cancer Patients.

genotypes	ER		*P* ^a^	*OR* (95%*CI*)^a^	PR		*P* ^a^	*OR* (95%*CI*)^a^	Her-2		*P* ^a^	*OR* (95%*CI*)^a^
	Negative (n = 149)	Positive (n = 293)			Negative (n = 188)	Positive (n = 253)			Negative (n = 120)	Positive (n = 308)		
rs6420545												
CC	40	78		1	55	63		1	36	77		1
CT	64	137	0.389	1.26 (0.75–2.13)	75	125	0.051	0.66 (0.99–2.76)	53	144	0.402	1.27(0.73–2.22)
TT	45	78	0.826	0.94 (0.53–1.65)	58	65	0.957	0.99(0.57–1.70)	31	87	0.313	1.38(0.74–2.58)
rs4801078												
CC	54	110		1	71	82		1	45	102		1
CT	61	123	0.770	1.08 (0.66–1.77)	75	109	0.145	1.43 (0.88–2.04)	46	134	0.478	1.22 (0.71–2.09)
TT	34	70	0.774	1.09(0.62–1.92)	42	62	0.370	1.28(0.74–2.21)	29	72	0.713	1.12(0.61–2.07)
rs1942348												
TT	59	110		1	75	94		1	48	117		1
CT	64	140	0.361	1.25 (0.78–2.01)	78	125	0.183	1.37 (0.86–2.16)	55	144	0.789	0.93 (0.56–1.57)
CC	26	43	0.761	0.91 (0.49–1.69)	35	34	0.344	0.75 (0.41–1.36)	17	47	0.723	0.88(0.45–1.75)
rs9636089												
TT	61	113		1	76	98		1	48	118		1
CT	63	137	0.301	1.28(0.80–2.05)	78	121	0.276	1.28(0.82–2.02)	55	142	0.936	0.98(0.59–1.63)
CC	25	43	0.932	1.03(0.55–1.92)	34	34	0.475	0.80(0.44–1.47)	17	48	0.935	1.03(0.51–2.06)

^a^*P* value of logistic regression analysis with adjusted for age, age at menarche, menopausal status, number of pregnancy and abortion, breast-feeding status, family history of BC in first-degree relatives.

### Haplotype analysis

The Haplotype analysis for polymorphisms in *Linc-ROR* was showed in Table 5.Four haplotypes were showed in the table (a total of 16 haplotypes) and all those frequencies < 0.03 in cases or controls has been dropped in this analysis. T_rs6420545_T_rs4801078_T_rs1942348_T_rs9636089_ was the most frequent haplotype in the cases (38.5%) and controls (35.3%). Compared with the controls, the frequency of haplotype T_rs6420545_C_rs4801078_T_rs1942348_T_rs9636089_ was lower in cases (*OR*: 0.72, *95%CI*: 0.54–0.97).

**Table 5:** Haplotype analysis of four SNPs in *Linc-ROR*.

Haplotype^a^	Cases (%)	Controls (%)	**χ** ^2^	P	OR (95%CI)
CCCC	318.36(0.33)	328.80(0.34)	0.028	0.867	0.98(0.81–1.19)
CCTT	96.25(0.10)	103.46(0.11)	0.143	0.705	0.95(0.70–1.27)
TCTT	87.43(0.09)	119.52(0.12)	4.87	**0**.**030**	**0**.**72(0**.**54–0**.**97)**
TTTT	372.34(0.39)	341.89(0.35)	3.44	0.060	1.20(0.99–1.45)

^a^SNPs sequence: rs6420545, rs4801078, rs1942348 and rs9636089.

The others haplotypes were ignored in analysis for the frequencies < 0.03.

### The analysis of Gene reproductive factors interactions

The interactions of gene and reproductive factors among four SNPs (rs6420545, rs4801078, rs1942348 and rs9636089) and reproductive factors were revealed in Table 6. Four models in MDR were showed and we found the interaction of rs4801078, number of pregnancy and menopausal status (T_rs4801078_, number of pregnancy ≥2 and post-menopausal) might increase risk for breast cancer by 2.78 times (*OR*: 2.78; *95%CI*: 2.74–3.61).

**Table 6:** Interaction results between the SNPs and reproductive factors by MDR.

Model	TBA^a^	CVC^b^	**χ** ^**2**^	P	OR(95%CI)
number of pregnancy	0.52	10/10	20.03	<0.01	1.81(1.39–2.35)
number of pregnancy, menopausal status	0.61	10/10	51.83	<0.01	2.57(1.98–3.33)
rs4801078, number of pregnancy, menopausal status	0.62	5/10	60.56	<0.01	2.78(2.74–3.61)
rs1942348, age at menarche, number of pregnancy	0.65	3/10	90.91	<0.01	3.73(2.82–4.92)

^a^Testing balance accuracy.

^b^Cross-validation consistency.

### The results of Benjamini-Hochberg (BH) correction for false discovery rate (FDR)

We applied the BH correction to control FDR (Table 7). The q-value indicated TT genotype and T allele of rs4801078in *Linc-ROR* could still increase BC risk. Besides, the relationship between CT + TT genotypes of rs4801078and BC was still noteworthy in the participant with age >50.

**Table 7:** Results of Benjamini-Hochberg (BH) correction.

Genotype		Stratified factors	*Adjusted P*	q-value
rs4801078	CC/TT	all subjects	0.005	**0**.**015**
	CC + CT/TT	all subjects	0.001	**0**.**006**
	C/T	all subjects	0.022	**0**.**044**
rs6420545	TT/CT + CC	age at menarche ≤ 13	0.005	0.070
	TT/CT + CC	no Breast-feeding	0.010	0.070
rs4801078	CC/CT + TT	age >50	0.002	**0**.**028**
	CC/CT + TT	age at menarche >13	0.025	0.175
	CC/CT + TT	no Breast-feeding	0.039	0.182
rs1942348	CC/CT + TT	age >50	0.045	0.630
rs9636089	TT/CT + CC	age >50	0.021	0.294

### The relative expression of *Linc-ROR* in plasma

**Figure 1:**
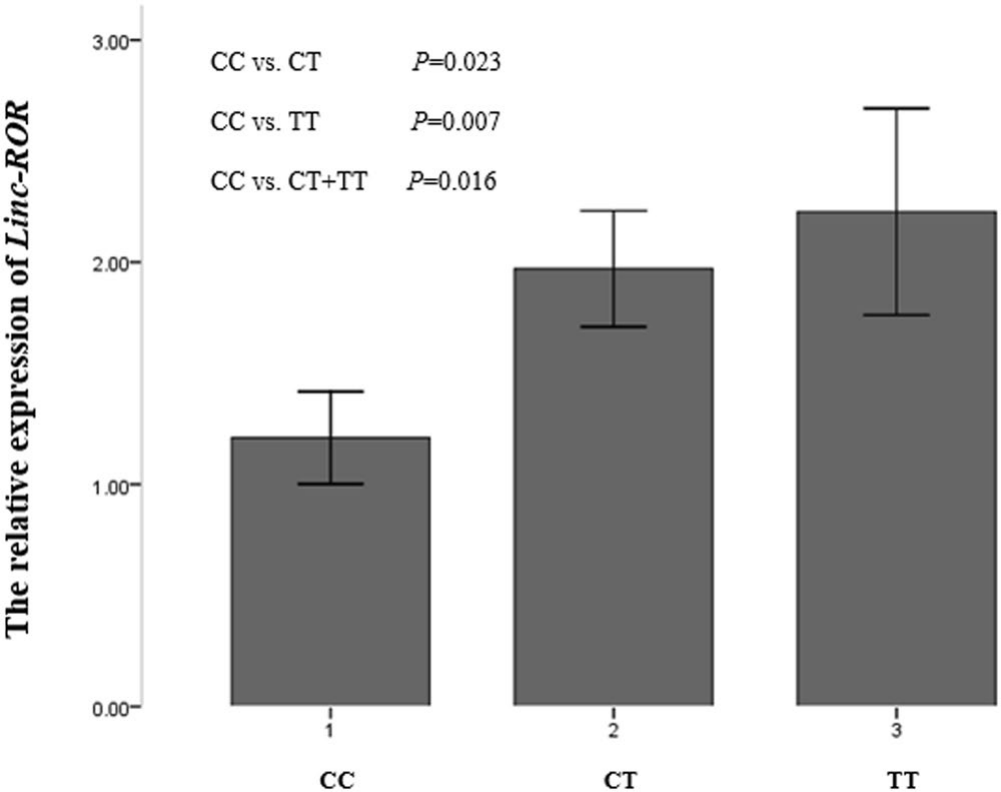
The relative expression of *linc-ROR* in plasma Note: *linc-ROR* expression was assessed by qRT-PCR in plasma. Data was evaluated statistically usingthe one-way ANOVA with the Tukey method and represent the mean ± SD from the experiments in triplicate.

We investigated the association between rs4801078 genotypes and *Linc-ROR* mRNA expression level by the real-time PCR amplification reactions in 150 subjects. There were 38 subjects with CC genotype, 64 with CT genotype and 48 with TT genotype (Fig. 1). The relative expression of *Linc-ROR* mRNA in CT + TT group (1.94 ± 0.27) was significantly higher than the CC group (1.21 ± 0.19) and that indicated the SNPs in *Linc-ROR* might play a role in the expression level of *Linc-ROR* mRNA.

## Discussion

This case-control study was the first to demonstrate the association between potential regulatory variants in *Linc-ROR* and BC risk, and we found TT (*OR*: 1.79; *95%CI*: 1.195–2.68) genotype of rs4801078 in *Linc-ROR* had a significant association with the higher risk of BC in Chinese population. Furthermore, analysis of qRT-PCR showed the relative expression of *Linc-ROR* mRNA in rs4801078CT + TT group (1.94 ± 0.27) was higher than the CC group (1.21 ± 0.19). In addition, we found the frequency of haplotype T_rs6420545_C_rs4801078_T_rs1942348_T_rs9636089_was lower in cases than in controls (*OR*: 0.72, *95%CI*: 0.54–0.97) and the interactions of rs4801078, number of pregnancy and menopausal status might increase BC risk (*OR*: 2.78; *95%CI*: 2.74–3.61).

LncRNAs have received widespread attention and are observed to play pivotal roles in tumorigenesis and progression of human cancers. It has already been revealed that some lncRNAs, such as HOTAIR, H19 and MALAT1, are potential biomarkers in cancer diagnosis and prognosis. Among them, *Linc-ROR*, first discovered in 2010, are also found to have strong association with tumorigenesis, metastasis and poor therapeutic response of malignant tumors^26–28^. Recent studies found that *Linc-ROR* was upregulated in pancreatic cancer tissues and decreased *Linc-ROR* expression could inhibit pancreatic cancer cell proliferation, invasion, and tumourigenicity^22,29,30^. In another study, the researchers found *Linc-ROR* confers gemcitabine resistance to pancreatic cancer cells at least partly via inducing autophagy^31^. Zhou *et al*. found that *Linc-ROR* had an important role during endometrial carcinogenesis by acting as a miR-145 “sponge” to inhibit mediation of the differentiation of endometrial tumorspheres^32^. A recent study suggested the function of *Linc-ROR* exerted in LAD cells depended on the sponging of miR-145 and it led to the chemotherapy resistance and EMT phenotypes of docetaxel-resistant LAD cells^33^. The qRT-PCR showed a significant up-regulation of *Linc-ROR* and its variants 2 (*P* = 0.025) and 4 (*P* = 0.0002) in esophageal squamous cell carcinoma^34^. Li *et al*. found that *Linc-ROR* was significantly upregulated in nasopharyngeal carcinoma tissues and the enrichment of *Linc-ROR* played acritical functional role in chemoresistance by suppressing P53 signal pathway^35^. A recent study also provided several new mechanistic insights into acquired chemoresistance in HCC and they found *Linc-ROR* acting as mediators are involved in modulation of cellular responses to chemotherapy^36^. However, the role of *Linc-ROR* in glioma is the opposite of other tumors. Feng *et al*. suggested that *Linc-ROR* might act as a novel tumor suppressor gene in glioma by inhibiting the proliferation of cancer cell, self-renewal of GSCs and the KLF4 expression^37^.

In addition to the mentioned malignant tumor, the role of *Linc-ROR* in breast cancer has also been reported. In 2014, Hou *et al*. found that *Linc-ROR* could function as an important regulator of epithelial-to-mesenchymal transition and promote breast cancer progression and metastasis through regulation of miRNAs^21^. In 2016, the study of Chen *et al*. investigated the role of *Linc-ROR* in the chemotherapy resistance of human BC cells and its mechanism^23^. The effect of the *Linc-ROR* on epithelial-to-mesenchymal transition was verified to contribute to the chemotherapy resistance and invasion of breast cancer cells^23^. In the same year, One study concluded that *Linc-ROR* suppressed gemcitabine-induced autophagy and apoptosis in breast cancer cells by silencing *miR-34a* expression^38^. Recently, Zhang *et al*. found down-regulated *Linc-ROR* could enhance the sensibility of breast cancer cells to tamoxifen by increasing miR-205 expression and suppressing the expressions of ZEB1 and ZEB2^39^. Zhao *et al*. suggested the expression levels of *Linc-ROR* were significantly higher in breast cancer and combination of the *Linc-ROR* with the conventional biomarkers might produce better diagnostic ability^40^. Together, these results indicate that *Linc-ROR* might have a crucial impact on the development of BC and it is necessary to investigate the association between regulatory variants in *Linc-ROR* and BC.

In recent years, a large amount of SNPs in lncRNAs have been found to be related to carcinogenesis^41^. For example, genetic variants in MALAT1A were suggested to be associated with breast cancer^42^ and colorectal cancer risk^27^. A combined analysis of genome-wide association study (GWAS) and meta-analysis identified a novel and significant association between rs16941835 in lncRNA*RP11-58A18*.*1* and CRC susceptibility^43^. Not only that, but those SNPs in lncRNAs were implied to have function in regulating the expression level of lncRNAs in the process of cancer. A genome-wide association study indicated that variant SNPs in a long noncoding RNA MIR2052HG showed a dose-dependent increase in MIR2052HG expression as well as increased binding of ERα to the EREs by performing estradiol treatment^44^. Another research showed the rs2147578 in lnc-LAMC2–1:1 were significantly associated with increased CRC risk by influencing the binding of lnc-LAMC2–1:1 and miR-128–3p^45^. In addition, the latest findings were reported that the SNP rs2027701 of *Linc-ROR* in the lncRNA-p53 regulatory network had significant associations with the risk of neutropaenia^42^. These results inspire us to study the role of *Linc-ROR* tagger SNPs in the expression of *Linc-ROR* mRNA and the the process of breast cancer.

However, to date, no study about the tagger SNPs of *Linc-ROR* has ever been reported in BC. In our case-control study, we found TT (*OR*: 1.79; *95%CI*: 1.20–2.68) genotype of rs4801078 in *Linc-ROR* had a significant association with the higher BC risk in codominant model (*OR*: 1.79; *95%CI*: 1.20–2.68) and recessive model (*OR*: 1.80, *95%CI*: 1.28–2.66). Moreover, the expression of *Linc-ROR* mRNA was closely related with the alleles of rs4801078. The results of qRT-PCR revealed the relative expression of *Linc-ROR* mRNA in rs4801078 CT + TT group (1.94 ± 0.27) was significantly higher than the CC group (1.21 ± 0.19). No meaningful association between the other three SNPs (rs1942348, rs6420545 and rs9636089) and the risk of BC was observed in the major models, however, women with the mutant alleles of rs1942348, rs6420545 and rs9636089 had a lower BC risk in the subgrounps with age at menarche ≤ 13 (*OR*: 0.95, *95%CI*: 0.92–0.99 for rs6420545) and age >50 (*OR*: 0.60, *95%CI*: 0.37–0.99 for rs1942348; *OR*: 0.57, *95%CI*: 0.35–0.92 for rs9636089). In addition, the Haplotype analysis showed haplotype T_rs6420545_C_rs4801078_T_rs1942348_T_rs9636089_ could decrease BC risk (*OR*: 0.72, *95%CI*: 0.54–0.97). Our results suggest that the regulatory polymorphisms in *Linc-ROR* might influence breast cancer risk and large sample studies carrying in other races are needed to verify our discovery.

BC is a complex disease and epidemiological studies suggest that the occurrence of BC is usually due to the gene-environmental factors interaction^46^. The reproductive factors have been proved to be related with BC, including menarche age, menopausal state, number of pregnancy, number of abortion, breast-feeding history for born baby^47,48^. In addition, a large number of SNPs in susceptibility genes have been found to be related to BC risk. There have been few reports of the interactions between SNPs in susceptibility genes and breast cancer influence factors^46,49^. However, the analysis of such gene-environment interaction is rarely reported in Chinese population and no study about the interaction between variants in *Linc-ROR* and the reproductive factors was found. In the current study, we found the interaction of rs4801078, number of pregnancy and menopausal status might increase BC risk by 2.78 times. We all know that reproductive factors usually represent a difference in hormone levels for women. Recent research suggested that *Linc-ROR* functions as an onco-lncRNA to promote estrogen-independent growth of ER+ breast cancer by the MAPK/ERK signaling pathway^24^. Moreover, another research showed the *Linc-ROR* expression levels in plasma were correlated with estrogen receptor (P < 0.05) and progesterone receptor (P < 0.05)^40^. Considering that the result of our study “functional polymorphisms in *Linc-ROR* might be associated with expression of *Linc-ROR*”, we assume that functional polymorphisms in *Linc-ROR* might have interaction with reproductive factors and the MDR analysis of our study proved the idea. Our results suggested that the gene-reproductive factors interaction might contribute to BC risk and further studies about the gene-reproductive factors interaction were warranted.

The strengths and limitations of the article are as follows: the new and pathological diagnosed BC patients avoids the prevalence-incidence bias; the selection bias is effectively controlled by random selection of the control group in the community; age and gender-matched controls and strict inclusion criteria reduce confounding bias; data was collected through the questionnaire by uniformly trained investigators and information was double entered in the database; moreover, randomly selected ten percent DNA samples were directly sequenced and each sample was repeated three times independently in qRT-PCR tests for the repeatability; nevertheless, in some subgroups, the statistical power is reduced due to the relatively small sample size; in addition, since early menarche was an established risk factor for breast cancer, the higher mean age at menarche in cases showed that there was still selection bias in our study.

To sum up, we found rs4801078 TT genotype in *Linc-ROR* had a significant association with the higher risk of BC and analysis of qRT -PCR showed the expression of *Linc-ROR* mRNA was closely related with the alleles of rs4801078. Rs4801078 might be related to BC by interacting with the number of pregnancy and menopausal status. Our results suggested that tagger SNPs in *Linc-ROR* and the interaction of tagger SNP-reproductive factors might contribute to BC risk, and the alleles of rs4801078 have positive correlation with *Linc-ROR* mRNA expression. Prospective studies with large sample and furthermechanism research are warranted to explore the role of the variants in *Linc-ROR*.

## Methods

### Subjects

A total of 968 participants were enrolled in the genetic epidemiology study between 2013 and 2015 from a community-based study in Henan province attended by 20000 individuals. Due to the low prevalence of breast cancer in the program, most of BC patients came from the First Affiliated and the Third Affiliated Hospital of Zhengzhou University. The inclusion criteria included the newly pathological diagnosed BC patients in Henan province without any other malignant tumor. 484 frequency matched cancer-free controls were selected from the program randomly. The inclusion criteria of the control group was healthy people without chronic diseases history and age-appropriate frequency matched (±2 years). All the participants were unrelated.

The demographic data and some potential BC risk factors were collected from a structured questionnaire by face to face interviews. The information of receptor status (estrogen receptor (ER), progesterone receptor (PR) and human epidermal growth factor receptor-2 (HER-2) status) came from pathological report. Some potential BC risk factors in the current study included age, age of menarche, menopause age, menopausal state (premenopausal, postmenopausal), gravidity, number of abortions, history of breast-feeding (yes, no) and BC family history in first-degree relatives (yes, no). Written informed consents were obtained from all participants. The study was approved by the ethical review committee of Zhengzhou University Committee for Medical and Health Research Ethics and all experiments were performed in accordance with relevant guidelines and regulations.

### DNA extraction

We collected 5 ml venous blood from each subject in ethylene diamine tetra acetic acid (EDTA), part of the blood cells were used to extract genomic DNA with the DNA Extraction Kit (TIANGEN BIOTECH, Beijing). Separated plasma and DNA samples were stored at −80 °C.

### SNP selection and genotyping

All tagger SNPs of *Linc-ROR* were selected by Haploview software basing on Chinese Han population data of HapMap Project (HapMapRel 28, NCBI B36) as well as 1000 Genome Project with minor allele frequency (MAF) > 0.1 in Chinese Han population (Table 8).

**Table 8:** PCR information of the four SNPs.

SNP ID	Allele	MAF	Genotyping assay	Tm(°C)	Primers(5′-3′)
rs1942348	T/C	0.44	CRS-RFLP	59.6	Sense: TTTCCCTCTTGGCTAATGCTGCTGA
					Antisense:TTACATAACTGTGGCAGAATGAAGG
rs6420545	C/T	0.45	PCR-RFLP	56.1	Sense: CTCCAGCCTAGATGACAGA
					Antisense: CACAGCAGCACTATTCCTAT
rs4801078	C/T	0.41	CRS-RFLP	56.1	Sense: ATTTCAAGCTCAGATCACTATAGAG
					Antisense: TCTAAGGGACAGAATAAATAATCGT
rs9636089	T/C	0.41	PCR-RFLP	59.6	Sense: GCACAGTTCACAGATGGA
					Antisense: CAGGAGATTGGCTTGGTT

rs6420545 and rs9636089were genotyped by polymerase chain reaction-restriction fragment-length polymorphism (PCR-RFLP) while the genotyping of rs1942348 and rs4801078were conducted by created restriction site PCR (CRS-RFLP) assays in our research. Primer 5.0 software was used to design the primer pairs of the four SNPs for PCR amplification and the NCBI BLAST was performed to evaluate the specificity of primers. Gradient PCR reactions was performed to standardize the DNA amplification conditions and optimize the annealing temperature for the primers set (Table 1). Rs6420545, rs9636089 rs1942348 and rs4801078 were digested by restriction enzymes of NsiI, MwoI, BlpI and BsmAI (Thermo Scientific) respectively which were selected by WATCUT website (http://watcut.uwaterloo.ca/watcut/watcut/template.php).In order to control the quality, we randomly selected 10% of the DNA samples to be sequenced (BGI Sequencing, Beijing).

### The relative expression of the *Linc-ROR* in plasma

One hundred and fifty individuals from the controls were selected and qRT-PCR test was used to detect the relative expression of *Linc-ROR* in plasma by the Eco Real-Time PCR System (Illumina, USA). The conditions for the reaction of real-time PCR are as follows: 1) initial denaturation: 95 °C for 30 s; 2) 40 cycles of denaturation: 95 °C for 10 s; 3) anneal: 60 °C for 30 s. Our assay provided GAPDH as a reference gene and the 2^−ΔΔCt^ method was performed to calculate the relative expression of *Linc-ROR*. The real-time PCR amplification of each sample was repeated three times.

### Analysis

The sample size (n = 459) of the study was calculated by the software power analysis and sample size (PASS)with the minimum allele frequency (0.25) and the study power (0.9). We assess the representativeness of the control population using a goodness-of-fit χ^2^-test (Hardy–Weinberg equilibrium).Categorical variables and continuous variables were calculated by Chi-squared and Student’s t test respectively to assess distribution departure in two groups. Unconditional logistic regression analysis was applied to estimate the relationship between SNPs and BC (or receptor status) with adjusted for the potential BC risk factors. Stratified analysis for the potential risk factors mentioned was made in different subgroups to estimate the relationship between SNPs and BC risk. MDR method was conducted to calculate the gene-reproductive factors interactions and online SHEsis (http://analysis.bio-x.cn/myAnalysis.php) was used to analyze the difference of haplotype frequencies in both patients and controls. Relative expressions of the gene *Linc-ROR* were presented as mean ± standard deviation ($\overline{X}$ ± SD) and the one-way ANOVA was applied to assess the difference. The Benjamini-Hochberg (BH) correction was used to control false discovery rate (FDR).The SPSS 21.0 statistical software package (Analysis software, Shanghai, co., LTD, 6761805c6989326cbf14) was used for all statistics analyses, and two-side *P* value less than 0.05 was regarded as significant.

### Data availability

The datasets generated during and analysed during the current study are available from the corresponding author on reasonable request.
